# Enhance the Er^3+^ Upconversion Luminescence by Constructing NaGdF_4_:Er^3+^@NaGdF_4_:Er^3+^ Active-Core/Active-Shell Nanocrystals

**DOI:** 10.1186/s11671-017-1929-8

**Published:** 2017-03-03

**Authors:** Xiaoyu Du, Xiangfu Wang, Lan Meng, Yanyan Bu, Xiaohong Yan

**Affiliations:** 1 College of Electronic Science and Engineering, Nanjing University of Posts and Telecommunications, Nanjing, 210046 People’s Republic of China; 2 Key Laboratory of Radio Frequency and Micro-Nano Electronics of Jiangsu Province, Nanjing, 210046 Jiangsu People’s Republic of China; 3 College of Science, Nanjing University of Aeronautics and Astronautics, Nanjing, 210016 People’s Republic of China

**Keywords:** Lanthanide doped, Active-core/active-shell, Upconversion, NaGdF_4_

## Abstract

NaGdF_4_:12%Er^3+^@NaGdF_4_:*x*%Er^3+^ (*x* = 0, 6, 8, 10, and 12) active-core/active-shell nanoparticles (NPs) were peculiarly synthesized via a delayed nucleation pathway with procedures. The phase, shape, and size of the resulting core–shell NPs are confirmed by transmission electron microscopy and X-ray diffraction. Coated with a NaGdF_4_:10%Er^3+^ active shell around the NaGdF_4_:12%Er^3+^ core NPs, a maximum luminescent enhancement of about 336 times higher than the NaGdF_4_:12%Er^3+^ core-only NPs was observed under the 1540 nm excitation. The intensity ratio of green to red was adjusted through the construction of the core–shell structure and the change of Er^3+^ concentration in the shell. By analyzing the lifetimes of emission bands and exploring the energy transition mechanism, the giant luminescence enhancement is mainly attributed to the significant increase in the near-infrared absorption at 1540 nm and efficient energy migration from the shell to core.

## Background

Upconversion (UC) refers to nonlinear optical processes characterized by the successive absorption of two or more pump photons via intermediate long-lived energy states, followed by the emission of the output radiation at a shorter wavelength than the pump one [1]. With the rapid development of nanotechnology, a large amount of high-quality lanthanide-doped upconversion nanoparticles (UCNPs) were synthesized and have been applied in various fields, such as solar cells [2, 3], bioimaging [4, 5], photo-catalysis [6], three-dimensional displays [7], and flash memories [8]. Especially for crystalline silicon solar cells, the adoption of UCNPs was proved to greatly improve the utilization of the solar energy. Nevertheless, the part (which wavelength is longer than 1100 nm) of solar energy will be lost for that the Si semiconductor cannot absorb the energy below its band gap (1.12 eV). And the propositon of UCNPs under the excitation of 1540 nm is an excellent way to reduce these sub band gap transmission losses. Thus, it becomes an important topic to utilize this part of solar energy more efficiently.

Moreover, the major challenge for UCNPs is still how to gain stronger upconversion luminescence with more optimized structures and synthesis methods. To overcome this challenge, many efforts were made, including changing the composition, tuning the morphology and size, and surface modification [9–17]. Among these methods, constructing core/shell architecture is thought to be the commonest but one of the most effective routes to improve the efficiency of UCNPs. By growing a shell around the luminescent core with similar lattice parameters, the lanthanide ions in the core were mostly protected from non-radiative decay caused by the surface defects. Besides, it is not until recent years that an active core/active shell structure is developed [9, 18–28]. Compared with traditional core/shell structure with an inert shell, an active shell not only protects the core from surface defects but also transfers absorbed near infrared (NIR) light from the pump source to the core NPs. In other words, the active shell acts as a sensitizer for the Ln^3+^-doped active core to increase the intensity of UC.

Commonly, an emissive UCNP consists of an inert host matrix and active Ln^3+^ ions as luminescent centers [29]. As an ideal activator, the ion tends to possess the ability of extracting energy from nearby excited sensitizers to promote further transitions to higher energy levels. Besides, to confine the process of non-radiative relaxations, the activator is likely to have energy levels separated to each other properly. According to the considerations above, Er^3+^, Tm^3+^, and Ho^3+^ are thought to be desired candidates. Moreover, Er^3+^ turns out to be the most effective one during the UC process, owing to its similar energy gaps. But, if simply increasing the concentration of Er^3+^ in the NPs, the luminescence shows a nonlinear relationship with the concentration of Er^3+^ and sometimes the luminescence intensity even decrease with the augment of Er^3+^, due to the influence of energy migration and concentration-quenching effects at higher concentrations.

Except successful construction of the active-core/active-shell structure, a strategy to improve the intensity of upconversion luminescence by only Er^3+^ ion-doped NaGdF_4_ was developed in this work. Compared to the inert shell, the UC luminescence was largely enhanced by adopting the method of the active shell and optimizing the Er^3+^ concentration. The luminescence properties of novel NaGdF_4_:12%Er^3+^@NaGdF_4_: 10%Er^3+^ active-core/active-shell NPs (Er^3+^ doping in mol% relative to Gd) were also discussed in detail under the 1540-nm excitation.

## Methods

### Materials

The starting materials were sodium trifluoroacetate (CF_3_COONa; reagent grade, 99%), trifluoroacetic acid (CF_3_COOH; reagent grade, 99%), gallium oxide (Gd_2_O_3_, 99.99%), and europium oxide (Er_2_O_3_, 99.99%). Cyclohexane (analytical grade, 99.5%), oleic acid (OA; analytical grade), oleylamine (OM; 80–90%), and absolute ethanol were used. Deionized water was used throughout. All chemical materials were used as received without further purification.

### Preparation of NaGdF_4_:12%Er^3+^ NPs

At the beginning, rare earth (RE) trifluoroacetate (RE(CF_3_COO)_3_; RE = Gd, Er) precursor was prepared as follows: rare earth oxides were dissolved in trifluoroacetic acid (CF_3_COOH) with certain amount of deionized water until the solution was transparent. The solution was then filtered, followed by drying at 140 °C for dozens of hours. The synthesis basically followed the routes previously reported in literature [30]. A mixture of a designated molar ratio of CF_3_COONa (1 mmol), Gd(CF_3_COO)_3_ (0.88 mmol), and Er(CF_3_COO)_3_ (0.12 mmol) powder were introduced to a three-necked flask (100 ml) containing 8 ml of OM and 16 ml of OA at room temperature. After vigorous stirring for about 15 min, the mixture was then heated to 120 °C under the protection of nitrogen or argon atmosphere and maintained at this temperature for another 30 min under magnetic stirring to remove the oxygen and residual water. At this end, the mixture was totally clear forming a slight yellow color. The mixture was then heated slowly to 275 °C in the presence of argon atmosphere and maintained at the temperature for 30 min. After then, the solution was cooled down naturally to room temperature. Finally, NPs were then precipitated using excess ethanol and collected via centrifugation at 7000 rpm for 5 min. After washed with ethanol for several times, the as-prepared nanocrystals were dried in air at 70 °C overnight.

### Preparation of NaGdF_4_:12%Er^3+^@ NaGdF_4_: *x*%Er^3+^ NPs

To prepare the NaGdF_4_:12%Er^3+^@NaGdF_4_:*x*%Er^3+^ core/shell UCNPs (*x* = 0, 6, 8, 10, and 12), core NPs of NaGdF_4_ doped with 12% Er^3+^ ion concentration were firstly prepared following the procedure as described above. The procedure for the growth of shell on core is similar to the case of core except that 0.5-mmol core NPs re-dispersed in 4 ml cyclohexane were added simultaneously with rare earth trifluoroacetates to the mixture of OA and OM solution and the heating temperature was enhanced to 300 °C.

### Characterization

Structures of the samples were investigated by X-ray diffraction (XRD) using X’TRA (Switzerland ARL) equipment provided with a Cu tube with Ka radiation at 1.54056 Å. The size and shape of the samples were observed by a JEM-2100 transmission electron microscope (JEOL Ltd., Tokyo, Japan). Luminescence spectra were obtained by the Acton SpectraPro Sp-2300 spectrophotometer with a photomultiplier tube equipped with 1540 nm as the excitation source. The fluorescence decay curves in visible region were recorded on a FLSP920 fluorescence spectrophotometer and using a Shimadzu R9287 photomultiplier (200–900 nm) as the detectors. All measurements were performed at room temperature.

## Results and Discussion

**Fig. 1:**
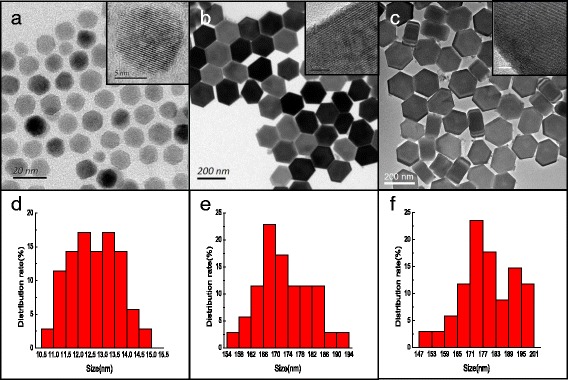
**a**–**c** TEM micrographic images and **d**–**f** size distribution of the NaGdF_4_:12%Er^3+^ core NPs, NaGdF_4_:12%Er^3+^@NaGdF_4_ active-core/inert-shell NPs and NaGdF_4_:12%Er^3+^@NaGdF_4_:10%Er^3+^ active-core/active-shell NPs, respectively

**Fig. 2:**
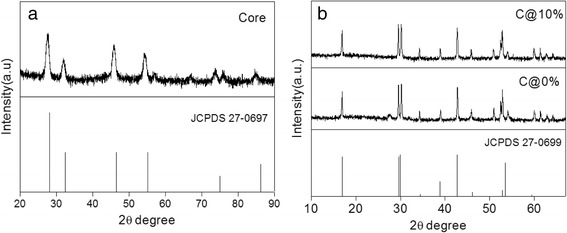
XRD patterns of the **a** NaGdF_4_:12%Er^3+^ core nanoparticles, **b** NaGdF_4_:12%Er^3+^@NaGdF_4_ active-core/inert-shell nanoparticles and NaGdF_4_:12%Er^3+^@NaGdF_4_:10%Er^3+^ active-core/active-shell nanoparticles (C@*x*% for short). The *bottom bars* represent the standard α-NaGdF_4_ (JCPDS 27–0697) and *β* − NaGdF_4_ crystal data (JCPDS 27–0699), respectively.

Figure 1 shows the transmission electron microscopy (TEM) images and the size distribution of the NaGdF_4_:12%Er^3+^ core NPs, NaGdF_4_:12%Er^3+^@NaGdF_4_ active-core/ inert-shell NPs, and NaGdF_4_:12%Er^3+^@NaGdF_4_:10%Er^3+^ active-core/active-shell NPs, respectively. Obviously, all the as-prepared NPs are uniform in size and morphology. The NaGdF_4_:12%Er^3+^ core-only NPs consist of monodisperse NPs with an average diameter of ~12.5 nm. After coated with a shell, the diameter of the NPs tends to increase to about 175 nm (Fig. 1b, c). As disclosed by high-resolution TEM (HRTEM) images (inset in Fig. 1), all as-prepared NPs show their highly crystalline nature with the clear crystal fringes. XRD patterns (Fig. 2a) confirm that as-obtained NaGdF_4_:12%Er^3+^ core NPs possess cubic phase structures, in well agreement with the α-NaGdF_4_ (JCPDS card no. 27−0697) structure. Coating with a shell, the NaGdF_4_:12%Er^3+^@NaGdF_4_ active-core/inert-shell NPs and the NaGdF_4_:12%Er^3+^@NaGdF_4_:10%Er^3+^ active-core/active-shell NPs show hexagonal phase, and the identified diffraction peaks are also in well agreement with the XRD pattern of *β*-NaGdF_4_ (JCPDS card no. 27−0699). No obvious peak from other phases or impurities is observed [31].

**Fig. 3:**
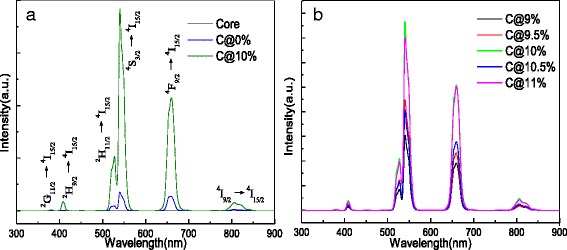
Upconversion emission spectra of **a** NaGdF_4_:12%Er^3+^ core NPs, NaGdF_4_:12%Er^3+^@NaGdF_4_ active-core/inert-shell NPs, and NaGdF_4_:12%Er^3+^@NaGdF_4_:10%Er^3+^ active-core/active-shell NPs. **b** The NaGdF_4_:12%Er^3+^@NaGdF_4_:*x*%Er^3+^ (*x* = 9, 9.5, 10, 10.5, and 11) active-core/active-shell NPs under 1540-nm excitation

**Fig. 4:**
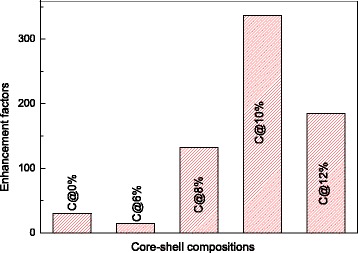
Enhancement factors of total emission intensities of NaGdF_4_:12%Er^3+^ core NPs, NaGdF_4_:12%Er^3+^@NaGdF_4_ active-core/inert-shell NPs, and the NaGdF_4_:12%Er^3+^@NaGdF_4_:*x*%Er^3+^(*x* = 6, 8, 10, and 12) active-core/active-shell NPs under 1540-nm excitation

Figure 3a shows the upconversion emission spectra of the core-only, active-core/ inert-shell, and active-core/active-shell NPs under the excitation at 1540 nm. All luminescence spectra exhibit the red, green, and NIR emission bands of Er^3+^, originating mainly from the following four transitions: ^2^H_11/2_→^4^I_15/2_ (528 nm), ^4^S_3/2_→^4^I_15/2_ (540 nm), ^4^F_9/2_→^4^I_15/2_ (660 nm), and ^4^I_9/2_→^4^I_15/2_ (810 nm). Compared with the NaGdF_4_:12%Er^3+^ core-only NPs and the NaGdF_4_:12%Er^3+^@NaGdF_4_ active-core /inert-shell NPs, great enhancement of upconversion luminescence was obviously observed from the NaGdF_4_:12%Er^3+^@NaGdF_4_:10%Er^3+^ NPs. As seen in Fig. 3b, the sample of NaGdF_4_:12%Er^3+^@NaGdF_4_:10%Er^3+^ NPs shows the strongest luminescence with shorter concentration step. Specifically, Fig. 4 presents the enhancement factors (which expresses the enhanced times) of all emission intensities of the NaGdF_4_:12%Er^3+^ core-only NPs, NaGdF_4_:12%Er^3+^@NaGdF_4_ active-core/ inert-shell NPs, and NaGdF_4_:12%Er^3+^@NaGdF_4_:*x*%Er^3+^(*x* = 6, 8, 10, and 12) active-core/active-shell NPs. Compared with the NaGdF_4_:12%Er^3+^ core-only NPs, the enhancement factor of the NaGdF_4_:12%Er^3+^@NaGdF_4_ active-core/inert-shell NPs is about 30 times larger. While when it comes to the NaGdF_4_:12%Er^3+^ @NaGdF_4_:10%Er^3+^ active-core/active-shell NPs, a largest enhancement factor up to approximately 336 is observed. Comparing the as-obtained NPs with same size, the upconversion luminescence tends to decrease firstly and then gets the largest enhancement at 10 mol% to ~336 times, so the energy transfer reaction plays the main role in the huge enhancement of luminescence other than the effect of size [23]. Apparently, the NPs with an active shell realize much more effective upconversion luminescence than that with an inert shell.

As is known, the emission intensity of NPs will be enhanced by coating an inert shell over them, due to the confine of surface passivation [32]. The inert shell protects the ions in the core from non-radiative decay originate from surface defects. Therefore, the NaGdF_4_:12%Er^3+^ NPs capped with NaGdF_4_ gain an enhancement factor about 30. However, the luminescence of the NPs tends to decrease first when doped the Er^3+^ ions with 6 mol%. As reported before, the decrease is mainly caused by the less efficiency of sensitizers in shell than that in the core [25]. The luminescence of NPs reaches the highest value when the concentration value in the shell is 10 mol%. Higher doped Er^3+^ concentration strengthened the function of the active shell, leading to more efficient energy migration from the shell to the core. When the concentration of the shell comes to 10 mol%, the interaction of the Er^3+^ ions in the core and the shell may reach the peak as observed, bringing the largest enhancement of luminescence to 336 times. Other values of doping concentration are not beneficial for the higher emission intensity enhancement, suggesting that the higher or lower doping concentration may lead to the cross relaxations (CR) or the reduction of the luminescent activators [27, 32].

**Fig. 5:**
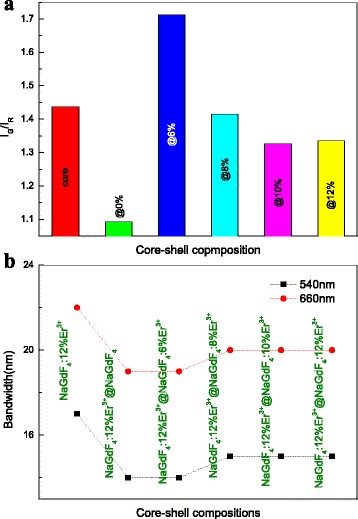
The effect of core–shell composition on **a** the intensity ratio between the green and the red emissions and **b** bandwidth values with the full width at half maximum of 540 and 660 nm

The effect of the shell structure on the intensity ratio of the green to red emission (I_G_/I_R_) is studied in Fig. 5a. With an inert shell, the value of I_G_/I_R_ decreases to ~1.09. The reduction of non-radiative decay (caused by suppression of surface quenching) perhaps increased the probability of the CR process of Er^3+^ ions, resulting in the enhanced population on ^4^F_9/2_ level [33]. It is apparent that the I_G_/I_R_ ratio is tuned by constructing the core–shell structure and changing the Er^3+^concentration in the shell. Essentially, the green and red emissions are mainly originated from the transitions of ^2^H_11/2_, ^4^S_3/2_→^4^I_15/2_, and ^4^F_9/2_→^4^I_15/2_ [34, 35]. Tuning the concentration of Er^3+^ in the shell directly changes the distance between the neighboring emitters, causing the cross relaxation to different extent [34]. And the emitter-doping concentration has been also verified to have a great influence in the ratio of green to red [36]. Consequently, it is easy to deduce that the as-observed changes of the green to red ratio of as-prepared NPs may mainly result from co-effect of concentration quenching and CR. And from the Fig. 5b, the bandwidth values of 540 and 660 nm about the NaGdF_4_:12%Er^3+^ core NPs, NaGdF_4_:12%Er^3+^@NaGdF_4_ active-core/inert-shell NPs, and NaGdF_4_:12%Er^3+^ @NaGdF_4_: *x*%Er^3+^(*x* = 6, 8, 10 and 12) active-core/active-shell NPs are presented. Coated with an inert or active shell, the bandwidth values with the full width at half maximum (FWHM) are all adjusted to lower values. The luminescent properties were well improved by tuning the FWHM values with core–shell structure.

Combined Fig. 3 with Fig. 5, one phenomenon can be found that the luminescence properties of as-obtained NPs exhibit an inhomogeneous increase mostly depending on the doping concentrations of Er^3+^ in the NaGdF_4_:Er^3+^ active shell. The phenomenon is also found in NaYF_4_:Nd^3+^/Yb^3+^/Ho^3+^@NaYF_4_:Nd^3+^/Yb^3+^ core/shell NPs under 808-nm excitation [27] and NaYF_4_:Er^3+^@NaYF_4_:Yb^3+^ active-core/active-shell NPs under 1540-nm excitation [28], probably owing to the combined interaction of energy transfer benefit, concentration quenching, and surface quenching effect of core–shell structures as estimation. When the concentration of Er^3+^ in the active shell comes to 10 mol%, the peak value can be observed through the luminescence spectra, largely due to the effect of successive ion layer absorption reaction.

**Fig. 6:**
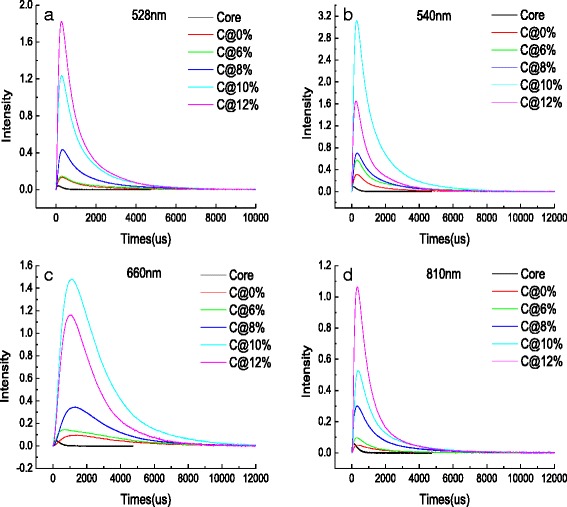
Decay profiles of **a** 528, **b** 540, **c** 660, and **d** 810 nm of the NaGdF_4_:12%Er^3+^ core NPs, the NaGdF_4_:12%Er^3+^@NaGdF_4_ active-core/inert-shell NPs, and the NaGdF_4_:12%Er^3+^@NaGdF_4_:*x*%Er^3+^(*x* = 6, 8, 10, and 12) active-core/active-shell NPs under 1540-nm excitation

As can be seen in Fig. 6, decay profiles of the NaGdF_4_:12%Er^3+^ core NPs, the NaGdF_4_:12%Er^3+^@NaGdF_4_ active-core/inert-shell NPs, and the NaGdF_4_:12%Er^3+^@ NaGdF_4_: *x*%Er^3+^ (x = 6, 8, 10, and 12) active-core/active-shell NPs under 1540-nm excitation are proposed. The corresponding decay lifetimes can be calculated by the second order exponential curve fitting:

1 $$ I={A}_1 \exp \left(- t/{\tau}_1\right)+{A}_2 \exp \left(- t/{\tau}_2\right) $$

where *I* is the luminescence intensity, *A*_*1*_ and *A*_*2*_ are constants, *t* is the time, and *τ*_*1*_ and *τ*_*2*_ are the short and long lifetimes for exponential components, respectively. The average decay lifetime *τ* is determined by the formula:

2 $$ \tau =\left({A}_1{\tau}_1^2+{A}_2{\tau}_2^2\right)/\left({A}_1{\tau}_1+{A}_2{\tau}_2\right) $$

**Fig. 7:**
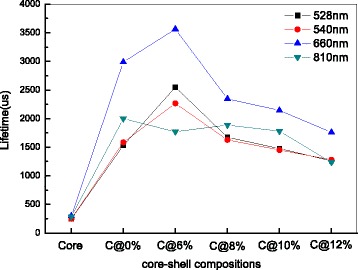
The calculation results of lifetimes of NaGdF_4_:12%Er^3+^ core NPs, NaGdF_4_:12%Er^3+^@NaGdF_4_ active-core/inert-shell NPs, and the NaGdF_4_:12%Er^3+^@NaGdF_4_:*x*%Er^3+^(*x* = 6, 8, 10, and 12) active-core/active-shell NPs under 1540-nm excitation

The results of the calculation are all shown in Fig. 7. As can be seen, the obvious prolonged life can be experimentally observed by coating with a shell, indicating that the excited states of ^2^H_11/2_ (528 nm), ^4^S_3/2_ (540 nm), ^4^F_9/2_ (660 nm), and ^4^I_9/2_ (810 nm) are populated again through the energy transfer process between Er^3+^ ions. Comparing the values of the decay lifetime about the as-obtained NPs, one can find that while continuing to increase the Er^3+^ concentration higher than 6 mol%, the lifetime begin to decrease. The phenomenon can be explained by the fact that the probability of CR process is largely related to the ions concentration [37]. The profiles of excited state ^2^H_11/2_, ^4^S_3/2_, and ^4^I_9/2_ appear no obvious signal rising edge, indicating that they are populated by non-radiative relaxation [38]. Contrary to other decay curves in Fig. 6, the profile of NaGdF_4_:12%Er^3+^@NaGdF_4_:10%Er^3+^ NPs at 660 nm presents a typical slow rising edge. This phenomenon is a direct evidence of an energy transfer upconversion process of state ^4^F_9/2_ [39]. As depicted in Fig. 7, the obvious prolonged lifetimes are experimentally observed at different degree, demonstrating that surface-quenching effects in both type of core/shell structure have been largely weaken. Comparably, the decay lifetimes of the NaGdF_4_:12%Er^3+^@NaGdF_4_ active-core/inert-shell NPs and the NaGdF_4_:12%Er^3+^@NaGdF_4_:10%Er^3+^ active-core/active-shell NPs under the 810 nm emission are smoother than other profiles, indicating that the population ions on the ^4^I_9/2_ state have less difference between different core–shell compositions.

**Fig. 8:**
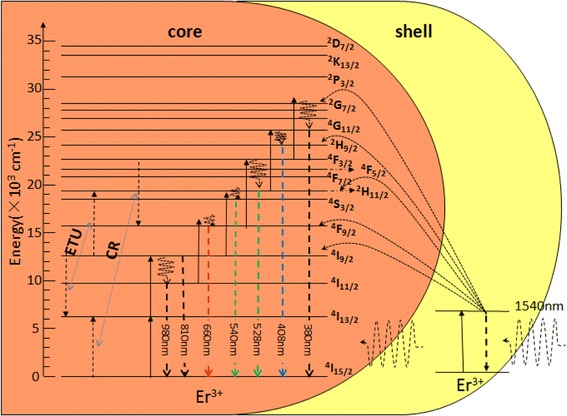
Proposed energy transfer mechanism in NaGdF_4_:12%Er^3+^@NaGdF_4_:10%Er^3+^ active-core/active-shell NPs under 1540-nm excitation

As analyzed above, the energy transfer mechanism between the states of Er^3+^ ions is exhibited in Fig. 8. Four primary processes can be mainly used to explain these behaviors. The ground state absorption (GSA) of the 1540-nm pump wavelength directly populates the upper level ^4^I_13/2_. With the excitation of ^4^I_13/2_ state, the excited state absorption (ESA) process arises immediately, resulting in more high-lying levels to be excited. After coating with an inert shell, the surface quenching is largely confined and the depletion of low-lying levels is reduced to enhance the population of high-lying ones, bringing intensive luminescence (as seen in Fig. 3). At the same time, the low-lying levels are proved to be more susceptible to surface quenching than the higher ones [32]. Thus, the emission of red luminescence has a more obviously enhancement than the green upon the surface passivation effect take place due to the coating of inert shell [40]. The ratio of green to red changes from 1.44 to 1.09 after coating with an inert shell. With the epitaxial growth of an active shell, the ratio of green to red changes by adjusting Er^3+^ concentration among the shell. The ratio shows an inverse association with the enhancement luminescence of active-core/active-shell NPs (Fig. 4). The phenomenon indicates that the CR process of ^4^F_3/2_ + ^4^I_15/2_→^4^F_9/2_ + ^4^I_13/2_ is strengthened with the enhancement luminescence faster than the process of energy transfer upconversion (ETU). From the decay profile of 810 nm, the lifetime of the as-obtained NPs decreases firstly while doping the Er^3+^ ions into the shell, which is another direct evidence of the ETU process, populating the ^4^S_3/2_ and ^2^H_11/2_ state from the ^4^I_9/2_ state.

By further studying the temporal behavior of the red emission, one can find that the decay curves of 660 nm of the NaGdF_4_:12%Er^3+^@NaGdF_4_ active-core/inert-shell NPs and the NaGdF_4_:12%Er^3+^@NaGdF_4_:10%Er^3+^ active-core/active-shell NPs exhibit a typical slow rising edge. As other work reported before, the rising edge is proved to be another evidence of CR as theoretical transients [39, 41]. Combined with the luminescence spectra, CR process of ^4^F_3/2_ + ^4^I_15/2_→^4^F_9/2_ + ^4^I_13/2_ makes large contribution to the population of the ^4^F_9/2_ state due to the higher and higher concentration of Er^3+^ in the shell, which also can be obtained from the calculated results of the lifetimes (Fig. 7). Besides, none emission of any state around ^4^F_3/2_ is found, proving the existence of CR. Consequently, under the co-effect of core/shell structure and optimized Er^3+^ concentration in shell, the energy transfer reaction is extremely improved, leading to the great luminescent enhancement of active-core/active-shell NPs.

## Conclusions

In conclusion, a strategy was put forward to enhance the upconversion luminescence by forming active-core/active-shell structure with Er^3+^ ions single-doped NaGdF_4_ without any other lanthanide-doped ions. Hexagonal NaGdF_4_:12%Er^3+^@NaGdF_4_:10%Er^3+^ active-core/active-shell NPs have been synthesized and present a significant intensive emission of the upconversion luminescence compared to NaGdF_4_:12%Er^3+^@NaGdF_4_ active-core/inert-shell and NaGdF_4_:12%Er^3+^ core-only NPs. Acting both as the emitter and the sensitizer, the luminescence of single Er^3+^-doped NaGdF_4_ UCNPs can be greatly enhanced by forming the structure of an active shell over the core. By the optimization of the concentration of Er^3+^ ions in the shell, the upconversion luminescent properties are greatly promoted and the enhancement of UC luminescence intensity is up to ~336 times greater than the NaGdF_4_:12%Er^3+^ core-only NPs, which is much larger than simply increasing the Er^3+^ concentration in NPs.

## Abbreviations

CR: Cross relaxation
ESA: Excited state absorption
ETU: Energy transfer upconversion
FWHM: Full width at half maximum
GSA: Ground state absorption
HRTEM: High-resolution TEM
NIR: Near infrared
NP: Nanoparticle
OA: Oleic acid
OM: Oleylamine
RE: Rare earth
TEM: Transmission electron microscopy
UC: Upconversion
XRD: X-ray diffraction
